# Auditory Cortex Morphology Predicts Language Learning Potential in Children and Teenagers

**DOI:** 10.3389/fnins.2019.00824

**Published:** 2019-08-07

**Authors:** Sabrina Turker, Susanne Maria Reiterer, Peter Schneider, Annemarie Seither-Preisler

**Affiliations:** ^1^Centre for Systematic Musicology, University of Graz, Graz, Austria; ^2^Department of Linguistics, University of Vienna, Vienna, Austria; ^3^Section of Biomagnetism, Department of Neuroradiology, Heidelberg University Hospital, Heidelberg, Germany; ^4^BioTechMed-Graz, University of Graz, Graz, Austria

**Keywords:** auditory cortex morphology, language aptitude, Heschl’s gyrus, foreign language learning, working memory, arithmetic fluency

## Abstract

In two recent studies, we identified neuroanatomical and neurofunctional markers of musical aptitude, attention deficit (hyperactivity) disorder and dyslexia in the auditory cortex (AC) of children. In a subsequent study with adults, we found evidence for neuroanatomical correlates of speech imitation ability in right Heschl‘s gyrus (HG), a structure comprising primary and parts of secondary AC. In the present study, we aimed to verify this previously suggested link between structural variation of right HG and language aptitude in a younger population of children and teenagers (*N* = 42; age range: 10–16 years), while behaviorally exploring the relationship between language aptitude, working memory, arithmetic skills and musicality. Behaviorally, scores on the language aptitude battery strongly correlated with working memory and speech imitation ability. Furthermore, we found that self- and parent-reported language aptitude and school grades were closely associated with language aptitude scores. Neuroanatomical analyses revealed a significant relationship between the occurrence of multiple HGs and high gray matter (GM) volumes in right AC and high language aptitude regardless of age, gender or musical ability. Additionally, low language aptitude was associated with the occurrence of single gyri in right AC. In accordance with previous research, we suggest that right HG might be associated with language aptitude, with a stronger gyrification and higher GM volumes being beneficial for successful auditory processing and the integration of speech-related cues.

## Introduction

An individual’s ability to acquire foreign languages varies considerably between different learners, notably due to differences in so-called language aptitude. According to Carroll (1990) and Gagné (2004, 2005), the term ‘language aptitude’ designates an individual’s innate potential to acquire new languages. It is thought to consist of several sub-components earlier defined by Carroll (1962, 1973, 1990); four sub-components) and more recently adapted by Skehan (1986, 2002; Dörnyei, 2014; three sub-components). Carroll (1962) suggested that the potential for learning foreign languages comprises outstanding phonetic coding ability (capacity to perceive, associate and retain sounds), associative memory (capacity to form links in memory), grammatical sensitivity (capacity to recognize and understand grammatical functions), and inductive learning ability (capacity to infer or induce rules of the structures of a language). Skehan (2002) integrated Carroll’s four components into the three stages of language processing: (1) phonetic coding (input processing), (2) grammatical analytic ability (central processing), and (3) memory retrieval (output processing). He further emphasized the large influence of working memory on each of these stages. Individuals with a high aptitude for learning foreign languages, thus, are expected to show high abilities in the aforementioned domains, while at the same time possessing high working memory capacity (for a theoretical overview, see Ameringer et al., 2018).

Language aptitude and its relationship with working memory, intelligence, arithmetic skills, and musicality have been at the core of intense research recently. Baddeley and Hitch (1974), Papagno et al. (1991), and Baddeley et al. (1998) were some of the first to suggest a strong link between parts of the working memory system and language aptitude, which has been supported and broadly discussed in later studies (Ellis and Sinclair, 1996; Baddeley, 2003a,b, 2017; Kormos and Sáfár, 2008; Wen and Skehan, 2011; Wen, 2012; Wen et al., 2017). Regarding intelligence and overall cognitive ability, it has been suggested that language aptitude might actually be nothing more than a spin-off of intelligence (see discussion in Ortega, 2014). However, Rimfeld et al. (2015) found that only one third of genetic influence in second language learning is shared with intelligence. Skehan (1998) and Sternberg (2002) suggest that the overlap between language aptitude and intelligence might simply stem from the fact that skills measured in language aptitude and intelligence tests are similar since language-relevant dimensions are assessed. Discussing the relationship between linguistic and arithmetic skills, Simmons and Singleton (2008) state that processes vital for reading, e.g., phonological processing, are equally important for mathematical skills. This, in turn, has fueled a discussion as to whether weaknesses in phonological processing could hinder the development of skills that rely on the manipulation and storage of verbal codes (e.g., counting, calculations), explaining the comorbidity between reading disorder and dyscalculia (Dirks et al., 2008; Simmons and Singleton, 2008; LeFevre et al., 2010). This is supported by Vukovic and Lesaux (2013), who linked verbal ability to arithmetic skills indirectly through symbolic numbering, arguing that general verbal ability is significant for children’s understanding and reasoning with numbers. The intricate link between language and music (Patel, 2003, 2012; Jackendoff, 2009; Jäncke, 2012) has been discussed in numerous studies (e.g., Milovanov et al., 2008; Dogil and Reiterer, 2009; Milovanov et al., 2009; Milovanov and Tervaniemi, 2011; Chobert and Besson, 2013; Vangehuchten et al., 2015), relating musical ability to vocabulary learning (Milovanov et al., 2008, 2009; Milovanov, 2009), the processing of non-native speech sounds (Slevc and Miyake, 2006; Kempe et al., 2015) and speech rhythm perception (Magne et al., 2016).

While numerous behavioral studies have explored the concept of foreign language aptitude, only few neurofunctional and -anatomical studies have investigated its underlying neurobiology. Regarding speech imitation and pronunciation ability, Dogil and Reiterer (2009), Reiterer et al. (2011), Hu et al. (2013), and Reiterer (2018) reported that structural and functional differences between highly and poorly skilled speakers were most pronounced in the left inferior parietal lobe around supramarginal gyrus, followed by left-hemispheric auditory areas. Looking at the neural basis of grammatical analytical ability, Kepinska et al. (2017a,b,c, 2018) found that highly skilled learners engaged more right-hemispheric frontal and parietal regions during novel grammar learning. Moreover, highly skilled learners in their study displayed different lateralization patterns in parietal and temporal areas and showed different functional connectivity in the right fronto-parietal tract. Mostly, however, research has not focused on specific brain region but has rather aimed at detecting differences in whole brain analyses.

Although AC is a vital area for language processing (Giraud et al., 2007), individual variations in its neuroanatomy and possible implications for the development of outstanding language skills have received little attention to date (for a methodological overview, see Marie and Golestani, 2017). So far, AC has been studied extensively with regard to functional and structural differences between left and right AC (McGettigan and Scott, 2012). While a “historically established one-size-fits-all view on lateralization of speech and language” (Flinker et al., 2019), has emphasized the importance of the left hemisphere and left AC for the perception of speech, there is emerging evidence that both hemispheres are indeed involved in speech perception and linguistic processing (Flinker et al., 2019). This has earlier also been confirmed by Reiterer et al. (2005), who provided evidence that right AC is essentially involved in the holistic processing of speech stimuli, and Poeppel et al. (2004), who reported robust bilateral AC activation to words and syllables. Quite recently, Sheng et al. (2018) confirmed a right lateralization for the processing of syllables in pre- and post-central superior temporal gyrus in an MEG study, highlighting again that both right and left AC are essential for the perception of speech. Concerning its relationship to language skills, Golestani and colleagues described specific morphological characteristics in the left AC of expert phoneticians, multiple or split left Heschl’s gyri (HG), which were not observed in unexperienced subjects (Golestani et al., 2011). In other studies, Golestani et al. (2002, 2007) related the neuroanatomy of AC to phonetical skills and expertise and found left-hemispheric white matter differences between fast and slow learners of foreign speech sounds (Golestani and Pallier, 2007; Golestani et al., 2007). In our previous study (Turker et al., 2017, 2018), we reported a direct link between right-hemispheric AC morphology, speech imitation and musicality in German-speaking adults. In particular, participants with higher scores in a speech imitation task and a musicality test, measuring rhythm and tonal perception, had significantly more complete posterior duplications in their right HG (i.e., it consisted of at least two complete gyri).

The major aim of the present study lies in the verification of this previously established relationship between language aptitude and AC morphology in a younger population of children and adolescents. According to the results of our previous study, we hypothesize that individuals with a high aptitude for learning foreign languages, as measured by a language aptitude battery, possess multiple gyri and display significantly higher gray matter (GM) volumes in right AC compared to those with poor scores on the aptitude tests. Moreover, we further aim to explore the intricate relationship between language aptitude, working memory, arithmetic skills and musicality in this younger group of participants and link their various abilities to school achievement.

## Materials and Methods

### Subjects

The correlational findings we present are from children who took part in a longitudinal study at the University Clinic Heidelberg (*N* = 64; *M*_age_ = 14.4 ± 1.1 years; 32 females). As we aimed to verify potential behavioral and neuroanatomical differences on a group-statistical level, we divided the participants into three equal groups according to the percentile ranks achieved in the LLAMA language aptitude battery (Meara, 2005). The cutoff points were set at the 33th and the 66th percentile, and the intermediate group was discarded from further group statistical analyses, resulting in a new sample of children (*N* = 42; *M*_age_ = 14.5 ± 1.3 years; 19 females). This new sample included the high aptitude group (*N* = 21, *M*_age_ = 14.6 ± 0.9 years; 9 females) and the low aptitude group (*N* = 21, *M*_age_ = 14.4 ± 1.4 years; 10 females).

All participants were healthy, monolingually raised, right-handed German native speakers between 10 and 16 years of age. They all had begun acquiring their first foreign language, namely English, when they were 10 ± 1 years old, and spoke between one (English) and three foreign languages (mostly French, Spanish, or Latin). Subjects with any history of neurological or psychiatric condition or learning disorder were excluded from the study. All children and their parents provided written informed consent before the experiment, as approved by the ethic commission of the Medical University Heidelberg. Participants received monetary reward for their participation.

### Testing Procedure

All children were tested during fixed weekends at the University Hospital Heidelberg, with a maximum of eight children participating in the behavioral testing and a structural MRI session per weekend. Each child completed both the behavioral testing (consisting of several online and paper-pencil tests on intelligence, musicality, language learning, working memory) and the MRI session on two consecutive days. Tests were conducted in pseudorandomized order.

### Behavioral Testing

#### Questionnaires and Interviews

Parents filled out a questionnaire developed by the authors of the present study, in which they reported the family’s socio-economic situation, their child’s school grades and the languages they were acquiring at that time. Furthermore, they were asked to give an indication (‘parent-reported aptitude’) how gifted they considered their child for acquiring foreign languages on a scale from 0 to 10 (0 equaling no potential at all, 10 equaling extremely high potential).

The children were interviewed in between the tests about their school grades in English and German and gave a concrete estimation of their potential for learning foreign languages (‘self-reported aptitude’; using an identical scale as the parents from 0 to 10). Children also provided details of their musical experience, stating how many instruments they were playing at the time of measurement, how many they had learnt (>6 months) in the course of their life, how much they linked singing and how well they could sing.

#### General Intelligence

The children’s non-verbal IQ was tested with the revised version of the Culture Fair Intelligence Test (CFT20-R; Weiß, 2008). The test did not include any language-related task. The four subtests ‘substitutions,’ ‘classifications,’ ‘matrices,’ and ‘reasoning’ are considered as measures of general intelligence (general fluid ability) according to the classical intelligence model of Cattell (1963). In the present study, age norms were used. According to the ICD-10 scheme (F70: IQ 50-69: mild intellectual disability) the cutoff criterion for exclusion from the study was an IQ < 70. As all subjects performed better than that, no one had to be excluded.

#### The LLAMA Language Aptitude Battery

Children performed the LLAMA language aptitude battery (Meara, 2005; Rogers et al., 2016) instead of the Modern Language Aptitude Test (MLAT; Carroll and Sapon, 1959) used in our previous study (Turker et al., 2017). The LLAMA language aptitude test is considered a measure of foreign language aptitude and consists of different sub-tests, which are loosely based on the MLAT and on related conceptions of language aptitude. The LLAMA language aptitude battery (see Table 1) was our preferable choice as it is available for free, easy to administer, language-independent and suitable for children. A recent study (Granena, 2018) has also shown that the LLAMA scores correlate with early learner’s attainment and that the two most recent language aptitude tests, Hi-LAB (Linck et al., 2013) and LLAMA both tap on the same constructs, that is explicit aptitude, implicit memory and implicit learning, thus supporting the validity of the tests.

**TABLE 1:** The four parts of the LLAMA language aptitude battery and details concerning the task being administered (Meara, 2005).

	**LLAMA B**	**LLAMA D**	**LLAMA E**	**LLAMA F**
Skill/Ability	Vocabulary Learning	Phonetic Memory	Sound-symbol correspondence	Grammatical inferencing
Test details	Learning of an unknown language (symbols combined with foreign words)	Recognition and memory of auditory presented words of an unknown language	Understanding and remembering of newly acquired sound-symbol combinations	Implicit understanding and application of the underlying structure of an unknown language

In the ‘Vocabulary learning’ sub-test (LLAMA B) children had 2 min to learn as many words associated with tiny figures as possible (only visual input). This sub-test assessed their ability to quickly form links in memory. The ‘Phonetic memory’ sub-test (LLAMA D) assessed the recognition of previously heard words in an unknown language. Participants were auditory presented with words of an unknown language one quickly after the other. After this auditory presentation, they were presented with one word at a time and had to decide for each stimulus if it was part of the sequence presented beforehand or not. In the ‘Sound-symbol correspondence’ sub-test (LLAMA E), children had 2 min to learn associations between 27 simple combinations of digits and letters (e.g., 0í) and consonant-vowel syllables (e.g., that 0í corresponds to the spoken syllable/pa/). In the testing phase, they were presented with auditory combinations of two pairs of digits and letters (e.g.,/patu/) and had to find the correct written form of these (e.g., 0í3é). Finally, in the ‘Grammatical inferencing’ sub-test (LLAMA F), children learnt the syntax and semantics of an unfamiliar language in 5 min by being provided with pictures and corresponding sentences. One picture always corresponded to one sentence that gives information about the syntax of the language and the meaning of elements in that sentence. After the learning phase, they saw the same pictures and completely new pictures and had to choose the grammatically correct sentence to describe the pictures. For the new pictures, they must have understood the semantic and syntactic rules of the language in order to be capable of choosing the correct sentence. Scores of the LLAMA were between 0 and 100%.

#### Speech Imitation Ability

The Hindi speech imitation test developed by Dogil and Reiterer (2009) and Reiterer et al. (2011) required participants to repeat four words and four sentences in Hindi, an unknown language to them. Participants were instructed to listen carefully via headphones and they heard each stimulus three times in a row. Thereafter, they were asked to repeat once what they have just heard. Native speakers of Hindi rated on a scale from 0 to 10, how well the participants managed to imitate the speech input (native-like ability; see Turker et al., 2017). In our study, native speakers of Hindi (*N* = 8; 4 females) were paid to rate the speech samples of all children in a separate online rating. To guarantee fairness, all samples were randomized during the rating and the sample the children had heard was always provided with their imitation attempts. We then assessed interrater reliability and calculated a Hindi score from the mean ratings. The Hindi test has been standardized with 140 adults on the basis of 30 raters by S. Reiterer and colleagues. Until now, the test has not been externally validated since comparable speech imitation tests are still lacking.

#### Musicality Assessment

For the assessment of musical aptitude, we applied the AMMA (Advanced Measures of Musical Audiation) by Gordon (1989), assessing the accuracy of tonal and rhythmic perception by comparing a given standard and a comparison melody that can be the same or slightly modified regarding pitch or rhythm. In each part of the AMMA, a maximum of 40 points could be achieved. The AMMA was externally validated by comparing performance to the Musical Aptitude Profile (MAP) developed by the same author (Hanson, 2019). Correlations of the different scales ranged from *r* = 0.7 – 0.8. Recently, the AMMA has been re-validated by the Goldsmiths Musical Sophistication Index (Gold-MSI; Müllensiefen et al., 2014) which assesses self-reported musical skills and behaviors on multiple dimensions in the general population. The correlations between the self-report inventory and the test scores of the AMMA were all in the range of 0.30–0.51, which is in the upper range of what is usually reported as the correlation between a ‘paper-based’ self-report measure and actual perceptual or cognitive ability tests.

#### Working Memory

In the verbal working memory test, subjects repeated digits, both forward and backward, and non-words. Digit stimuli for both the forward and backward task were taken from the KAI (Lehrl et al., 1992). In the non-word repetition task, subjects needed to repeat German non-words that were created from a syllable database developed according to German phonotactic rules (e.g., “knoll,” “pflax,” “bamp”) at the Institute of Natural Language Processing, University of Stuttgart (Benner, 2005). For both tasks, each participant had two chances to correctly repeat a certain number of digits/non-words before another element was added. For each of the three tasks, one correct repetition yielded one point and a total of 14 points could be achieved in each of the separate tasks (maximum for all three tasks: 42 points).

#### Arithmetic Competence

The arithmetic fluency test we applied was based on the French Kit test of arithmetic skills (French et al., 1963; described in Vogel et al., 2017). The test included three types of calculations, namely additions, subtractions and multiplications. The test measured how many calculations children could perform (correctly) in a given time frame. For each correct calculation, participants received 1 point and points were added up for each page. Children were instructed to open the booklet, wait for the start signal and always start with the calculations at the top of each column and not skip any in between.

### Neuroanatomical Analyses

A T1-weighted structural magnetic MRI (Siemens, TrioTim, 3 Tesla) was performed to investigate the anatomy of AC. A standardized individual approach of three-dimensional GM surface reconstruction of auditory subareas (HG, planum temporale/PT) was applied to account for individual morphology and gyrification patterns (Schneider et al., 2002, 2005; Wengenroth et al., 2013; Seither-Preisler et al., 2014; Serrallach et al., 2016; Benner et al., 2017; Zoellner et al., 2018).

For segmentation, Brain Voyager software QX 2.8 (Brain Innovation, B.V, Maastricht, Netherlands) was used. All brain images were adjusted in contrast and brightness, precisely corrected for inhomogeneity and rotated in direction of the antero-posterior commissural line (Talairach and Tournoux, 1988). The superior temporal plane, including HG, the anterior superior temporal gyrus (aSTG) and PT, was segmented into sagittal MRI slices along the Sylvian fissure. This was done by using the standard definition of the landmarks of AC and approved additional criteria: the first complete Heschl’s sulcus with a large mediolateral extent (>97%) and pronounced depth was used as the posterior boundary in the case of single HGs and common stem duplication (CSD), and the last complete Heschl’s sulcus in the case of complete posterior or multiple HG duplications. The crescent-shaped first transverse sulcus was used as the anterior boundary of HG, thereby dividing AC into two parts, namely (1) an anterior stream including HG, HG duplications and aSTG and (2) a posterior stream including PT. HG was separated from aSTG by an anterior borderline with *y* = 0 (Schneider et al., 2005; Wengenroth et al., 2010; Seither-Preisler et al., 2014; Serrallach et al., 2016). The range of the included image gray values was calculated individually. A box was marked around left and right AC to generate intensity histograms of these areas. The ‘gray value inclusion range,’ which was used for surface reconstruction and morphometry, was defined on the basis of two criteria: (1) the value of the GM peak multiplied by the factor 0.28, which characterizes an appropriate cutoff value to separate liquor from GM tissue, (2) the saddle point between gray and white matter peaks. The gray and white value voxels embedded in this inclusion range were marked and used for 3D reconstruction; for morphometry only GM values were used.

**FIGURE 1:**
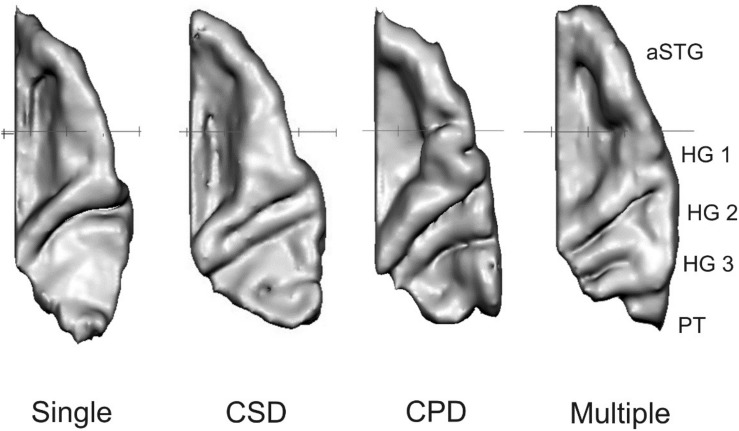
The four different types of HGs: single gyrus (single), common stem duplication (CSD), complete posterior duplication (CPD) and multiple gyri (multiple) from left to right. HG, Heschl’s gyrus; PT, planum temporale; aSTG, anterior superior temporal gyrus.

In our previous study (Turker et al., 2017) we distinguished between three types of HGs, namely single gyri (single), CSD and complete posterior duplications (CPD; including multiple gyri). In other studies, such as Benner et al. (2017), at least four types of gyri were distinguished. These were the three aforementioned and multiple gyri (at least three complete HGs). Due to the larger variability in HG types in the present study and the larger sample size (*N* = 42), we used this fourfold categorization this time. Therefore, all HGs for each hemisphere were separately classified by two members of our research group and then placed in the appropriate category (see Figure 1). In rare cases where classifications by the two researchers did not match, the specific cases were discussed and one classification was agreed on.

## Results

### Behavioral Results

To guarantee that potential differences on the scales of interest are not caused by differences in intelligence and socio-economic status, we initially compared the two aptitude groups for the age-normalized IQ achieved in the CFT20-R (Weiß, 2008) and measures of socio-economic background (Schneider and Seither-Preisler, 2015). The latter were determined from a general questionnaire for parents, developed by the authors of this study. A principal components analysis revealed three relevant social dimensions: (1) education environment (including the mother’s and father’s highest professional degree and the number of books at home); (2) parental engagement (including the amount of parent–child communication, the frequency of common participation in cultural events, and the parents’ personal interest in children’s activities); and (3) resources and leisure activities (including courses in sports, arts, etc., and children’s resources, such as their own room, personal computer, etc.). Parental income loaded as well on factors 1 and 3. The individual factor scores on each dimension (which are indirect measures and therefore not reported as descriptive statistics in the following), were compared across groups. Children in the two groups neither differed in IQ [low aptitude group: *M* = 107 ± 16.2; high aptitude group: *M* = 114.4 ± 12.8; *t*_(__38__)_ = −1.57, *p* = 0.126], nor in socio-economic status [education environment: *t*_(__38__)_ = −1.96, *p* = 0.06, parental engagement: *t*_(__38__)_ = −0.29, *p* = 0.77, resources and leisure activities: *t*_(__38__)_ = 0.85, *p* = 0.40]. Furthermore, we verified that age did not show significant correlations with any of the variables of interest and it was therefore not used as a covariate in further statistical analyses.

#### Correlational Analyses

First, a correlational analysis was performed for all assessed scales in all tested children (*N* = 64). Spearman’s rank correlation coefficients (Spearman’s ρ) and *p*-values of related scales are displayed in Table 2. Results of the AMMA test are not reported since there was only one significant correlation between AMMA tonal perception and digit span backward.

**TABLE 2:** Spearman correlation matrix for all assessed scales.

	**Languages**	**SR aptitude**	**PR aptitude**	**German Grade**	**English Grade**	**Hindi Score**	**Digit span forward**	**Digit span backward**	**Non-word span**	**LLAMA B**	**LLAMA D**	**LLAMA E**	**LLAMA F**	**Arithmetic skills**
Languages	1	*r* = 0.374^*^ ***p* = 0.002**	*r* = 0.379^*^ ***p* = 0.004**	*r* = −0.246 *p* = 0.095	*r* = −0.261^*^ *p* = 0.040	*r* = 0.207 *p* = 0.104	*r* = 0.298^*^ *p* = 0.018	*r* = 0.078 *p* = 0.549	*r* = 0.234 *p* = 0.068	*r* = 0.088 *p* = 0.494	*r* = 0.364^*^ ***p* = 0.003**	*r* = 0.314^*^ ***p* = 0.012**	*r* = 0.254^*^ *p* = 0.045	*r* = 0.251 *p* = 0.057
SR aptitude		1	*r* = 0.787^*^ ***p* < 0.001**	***r* =** −0.407^*^ ***p* = 0.005**	***r* =** −0.592^*^ ***p* = < 0.001**	*r* = 0.082 *p* = 0.523	*r* = 0.340^*^ ***p* = 0.006**	*r* = 0.273^*^ *p* = 0.032	*r* = 0.059 *p* = 0.650	*r* = 0.116 *p* = 0.367	*r* = 0.265^*^ *p* = 0.035	*r* = 0.214 *p* = 0.093	*r* = 0.248^*^ *p* = 0.050	*r* = 0.183 *p* = 0.170
PR aptitude			1	*r* = −0.364^*^ *p* = 0.025	***r* =** −0.592^*^ ***p* < 0.001**	*r* = 0.120 *p* = 0.388	*r* = 0.475^*^ ***p* < 0.001**	*r* = 0.248 *p* = 0.071	*r* = 0.176 *p* = 0.203	*r* = 0.164 *p* = 0.231	*r* = 0.225 *p* = 0.099	*r* = 0.245 *p* = 0.072	*r* = 0.393^*^ ***p* = 0.003**	*r* = 0.175 *p* = 0.223
German grade				1	*r* = 0.596^*^ ***p* < 0.001**	*r* = −0.280 *p* = 0.060	*r* = −0.230 *p* = 0.119	*r* = −0.169 *p* = 0.261	*r* = −0.101 *p* = 0.506	*r* = −0.240 *p* = 0.109	*r* = −0.171 *p* = 0.255	*r* = −0.198 *p* = 0.187	*r* = −0.195 *p* = 0.194	*r* = −0.199 *p* = 0.212
English grade					1	*r* = −0.042 *p* = 0.749	*r* = −0.165 *p* = 0.204	*r* = −0.262^*^ *p* = 0.043	*r* = −0.049 *p* = 0.710	*r* = −0.179 *p* = 0.168	*r* = −0.235 *p* = 0.069	*r* = −0.224 *p* = 0.082	*r* = −0.170 *p* = 0.192	*r* = −0.100 *p* = 0.465
Hindi score						1	*r* = 0.234 *p* = 0.067	*r* < −0.001 *p* = 0.998	*r* = 0.196 *p* = 0.130	*r* = 0.323^*^ ***p* = 0.009**	*r* = 0.145 *p* = 0.260	*r* = 0.201 *p* = 0.118	*r* = 0.152 *p* = 0.238	*r* = 0.113 *p* = 0.403
Digit span forward							1	*r* = 0.524^*^ ***p* < 0.001**	*r* = 0.519^*^ ***p* < 0.001**	*r* = 0.261^*^ *p* = 0.040	*r* = 0.379^*^ ***p* = 0.002**	*r* = 0.405^*^ ***p* = 0.001**	*r* = 0.296^*^ *p* = 0.020	*r* = 0.373^*^ ***p* = 0.004**
Digit span backward								1	*r* = 0.198 *p* = 0.123	*r* = 0.288^*^ *p* = 0.025	*r* = 0.315^*^ *p* = 0.014	*r* = 0.379^*^ ***p* = 0.003**	*r* = 0.166 *p* = 0.200	*r* = 0.458^*^ ***p* < 0.001**
Non-word span									1	*r* = 0.107 *p* = 0.413	*r* = 0.243 *p* = 0.059	*r* = 0.164 *p* = 0.208	*r* = 0.275^*^ *p* = 0.032	*r* = 0.006 *p* = 0.966
LLAMA B										1	*r* = 0.268^*^ *p* = 0.033	*r* = 0.318^*^ ***p* = 0.011**	*r* = 0.050 *p* = 0.697	*r* = 0.394^*^ ***p* = 0.002**
LLAMA D											1	*r* = 0.276^*^ *p* = 0.029	*r* = 0.080 *p* = 0.532	*r* = 0.250 *p* = 0.058
LLAMA E												1	*r* = 0.277^*^ *p* = 0.028	*r* = 0.299^*^ *p* = 0.023
LLAMA F													1	*r* = 0.209 *p* = 0.115
Arithmetic skills														1

Correlation coefficients (*r*) and significance values (*p*) are provided for each correlation, rounded to three decimal places. ^*^Significant at *p* < 0.05 before correction; bold = significant at *p* < 0.05 after correction [Benjamini–Hochberg correction for multiple comparisons (Benjamini and Hochberg, 1995) performed in R (p.adjust function]; Languages, number of languages; SR, self-reported; PR, parent-reported; arithm., arithmetic.

The number of foreign languages learnt by a child correlated positively with self-reported (*r* = 0.374, *p* = 0.002) and parent-reported aptitude (*r* = 0.379, *p* = 0.004), and with two measures of the language aptitude battery, namely phonetic memory (*r* = 0.364, *p* = 0.003) and sound-symbol association (*r* = 0.314, *p* = 0.012). Self-reported aptitude showed a high correlation with parent-reported aptitude (*r* = 0.787, *p* < 0.001) and school grades in German (*r* = −0.407, *p* = 0.005) and English (*r* = −0.592, *p* < 0.001). Furthermore, self-reported aptitude correlated with digit span forward (*r* = 0.340, *p* = 0.006), while parent-reported aptitude also correlated with English grade (*r* = −0.592, *p* < 0.001; German grade *p*-values did not survive statistical correction) and digit span forward (*r* = 0.475, *p* < 0.001). Parent-reported aptitude was further linked to LLAMA F, measuring grammatical inferencing (*r* = 0.393, *p* = 0.003). Moreover, German and English grades correlated highly with each other (*r* = 0.596, *p* = 0.001).

The Hindi score, considered a measure of non-word span, was only linked to vocabulary learning (LLAMA B, *r* = 0.323, *p* = 0.009). Digit span forward, on the other hand, was not only strongly associated with digit span backward (*r* = 0.524, *p* < 0.001) and non-word span (*r* = 0.519, *p* < 0.001), but it correlated with two language aptitude scores (LLAMA D: *r* = 0.379, *p* = 0.002; LLAMA E: *r* = 0.405, *p* = 0.001). Digit span backward was not related to non-word span, but to LLAMA E (*r* = 0.379, *p* = 0.003). There was a link between LLAMA B and E (vocabulary learning, sound-symbol association; *r* = 0.318, *p* = 0.011). Arithmetic skills correlated highly with digit span forward (*r* = 0.373, *p* = 0.004), digit span backward (*r* = 0.458, *p* < 0.001) and vocabulary learning (LLAMA B, *r* = 0.394, *p* = 0.002).

#### Principal Component Analysis

We performed a principal component analysis (PCA) for the different sub-scores of all tests (divisions, subtractions, additions, and multiplications belonging to arithmetic competence; digit span forward, backward and non-word span belonging to working memory; LLAMA B-E and Hindi belonging to the language aptitude score; singing ability, singing passion, AMMA rhythm, AMMA tonal and number of instruments belonging to musicality). A major aim of the PCA was to see whether working memory scores construct their own entity and should be considered separately from language aptitude (see Table 3).

**TABLE 3:** Results of the PCA analyses.

	**Rotated Component Matrix**		
	**Mathematical abilities**	**Language aptitude/Working memory**	**Musical ability**
Divisions (AC)	0.914		
Subtractions (AC)	0.909		
Additions (AC)	0.888		
Multiplications (AC)	0.820		
Digit span forward		0.787	
Digit span backward	0.367	0.562	
Non-word span		0.675	
LLAMA E		0.618	
LLAMA B		0.552	
LLAMA F		0.437	
LLAMA D		0.478	
Hindi		0.493	
Singing ability			0.825
Singing passion			0.672
AMMA rhythm			0.596
AMMA tonal			0.574
Number of instruments			0.545

*Extraction method: principal component analysis. Rotation method: varimax with kaiser normalization. Three components were identified, which confirm the assumed independence of the dimensions ‘arithmetic skills,’ ‘language aptitude,’ and ‘musical ability,’ with language aptitude being closely related to the three scales of working memory capacity.*

From the 17 variables included in the analysis, three core components could be extracted. The first component includes all tasks related to mathematic or arithmetic abilities. Additionally, digit span forward also loaded moderately on this factor. The second factor summarizes all tasks measuring language aptitude and working memory capacity, thereby confirming previous findings and our hypothesis that language aptitude is inextricably linked to working memory capacity. The variables loading most heavily on this factor are digit span forward, non-word span, LLAMA E, LLAMA B and digit span backward. The last factor summarizes all variables loading heavily on musical ability, namely singing ability, singing passion, AMMA scores and the number of instruments ever learnt. The findings of the PCA suggest that three very separate concepts were measured in our study: mathematical ability, language aptitude/working memory, and musicality.

#### Comparison Between Non-gifted and Gifted Learners

**FIGURE 2:**
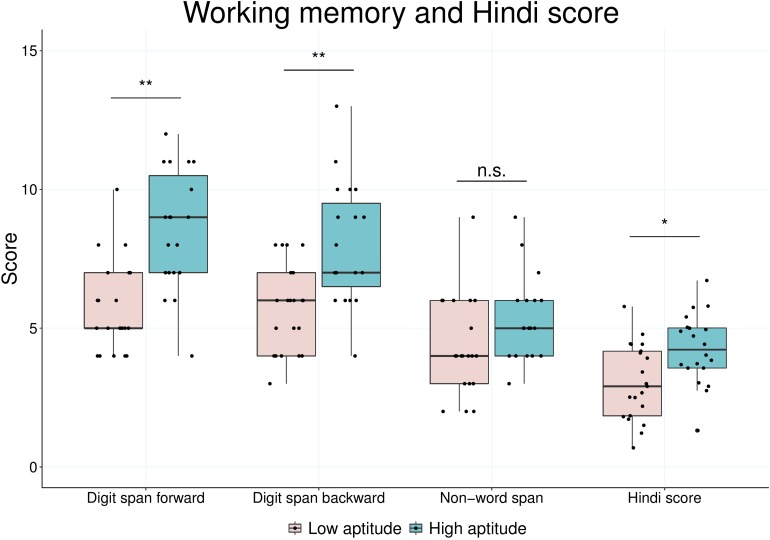
Comparison of children with low (left) and high (right) language aptitude regarding performance in digit span forward, digit span backward and non-word span, as well as Hindi speech imitation. ^∗∗^*p* < 0.001, ^*^p < 0.05.

In Table 4, differences between high-aptitude and low-aptitude individuals are shown for several measured scales. Subjects in the high-aptitude group were not only learning more languages (*Z* = −3.13, *p* = 0.002), but also had higher self-reported (*Z* = −3.19, *p* = 0.001) and parent-reported aptitude (*Z* = −3.07, *p* = 0.002). Moreover, their grades in language subjects at school were significantly better, most evidently in English (*Z* = −*2.56*, *p* = 0.010), but also in German (*Z* = −2.29, *p* = 0.022). Children with high language aptitude scores also had significantly better results in speech imitation (*Z* = −2.77, *p* = 0.006), two of the three working memory tasks (digit span forward: *Z* = −3.87, *p* < 0.001; digit span backward: *Z* = −3.27, *p* = 0.001) and better arithmetic skills (*Z* = −2.64, *p* = 0.008). Results are graphically displayed in Figure 2, showing the comparison of high- and low-aptitude children on working memory tasks and the Hindi test. To summarize, all measured scales could be linked to the distinction between above average and below average language aptitude, although there was no significant difference in intelligence between the two groups.

**TABLE 4:** High aptitude children score significantly higher on assessed scales than children in the low aptitude group (results of Mann–Whitney *U* Test).

	**High aptitude *N* = 21**	**Low aptitude *N* = 21**	***Z*-value**	**Significance**	***r***
Number of foreign languages	2.4 ± 0.7	1.7 ± 0.6	−3.13	*p* = 0.002	0.48
Self-reported aptitude	6.9 ± 1.1	5.6 ± 1.3	−3.19	*p* = 0.001	0.49
Parent-reported aptitude	7.7 ± 1.9	5.5 ± 1.7	−3.07	*p* = 0.002	0.47
School grade English	2.0 ± 0.8	2.8 ± 1.1	−2.56	*p* = 0.010	0.40
School grade German	2.1 ± 0.7	2.7 ± 0.7	−3.07	*p* = 0.002	0.47
Hindi score	4.3 ± 1.2	3.1 ± 1.4	−2.77	*p* = 0.006	0.43
Digit span forward	8.5 ± 2.1	5.8 ± 1.6	−3.87	*p* < 0.001	0.60
Digit span backward	8.0 ± 2.2	5.7 ± 1.6	−3.27	*p* = 0.001	0.51
Non-word span	5.3 ± 1.5	4.3 ± 1.7	−1.90	*p* = 0.058, n.s.	0.29
Arithmetic score	158.5 ± 34.5	123.6 ± 41.7	−2.64	*p* = 0.008	0.41

**Z*- and *p-*values, as well as effect size [correlation coefficient *r*, z-value divided by the square root of the total sample size (Field, 2013)] are displayed.*

### Neuroanatomical Results

**FIGURE 3:**
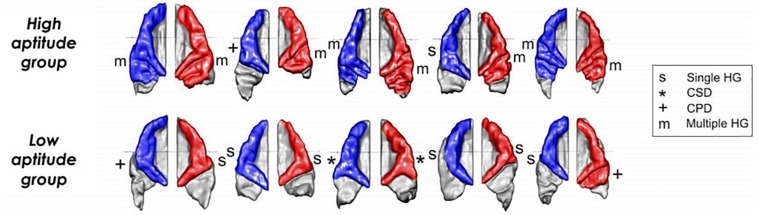
Examples of individual auditory cortices of the high **(first row)** and low language aptitude groups **(second row)** with type of HG indicated for each hemisphere. Left hemisphere: blue; right hemisphere: red.

In each AC, left and right HGs were manually segmented, morphologically classified according to the predefined types and analyzed with regard to GM volumes. In a next step, the frequency distributions of the four HG types were calculated (Table 5). Similar to the results found in our previous study (Turker et al., 2017) the left hemisphere showed slightly less variation compared to the right hemisphere. In the right hemisphere, the predominant HG type was multiple (*N* = 17), followed by single (*N* = 15), CPD (*N* = 7) and CS [*N* = 3; χ^2^_df = 3__,_ *_N_*_= 42_ = 12.5, *p* = 0.006). In contrast, in the left hemisphere, the predominant HG type was single (*N* = 20), followed by CPD (*N* = 12), multiple (*N* = 6) and CS (*N* = 4); χ^2^_df = 3__,_ *_N_*_= 42_ = 14.7, *p* = 0.002. Four exemplary high- and low-aptitude children’s auditory cortices are displayed in Figure 3.

**TABLE 5:** Distribution of HG types, namely single gyrus (single), common stem duplication (CSD), complete posterior duplication (CPD), and multiple gyri (multiple) in both hemispheres.

**Types of HG**	**Number in LH (%)**	**Number in RH (%)**
Single	20 (47.6%)	14 (33.33%)
CSD	4 (9.5%)	4 (9.5%)
CPD	12 (28.6%)	7 (16.66%)
Multiple	6 (14.3%)	17 (40.5%)
Total	42 (100%)	42 (100%)

**FIGURE 4:**
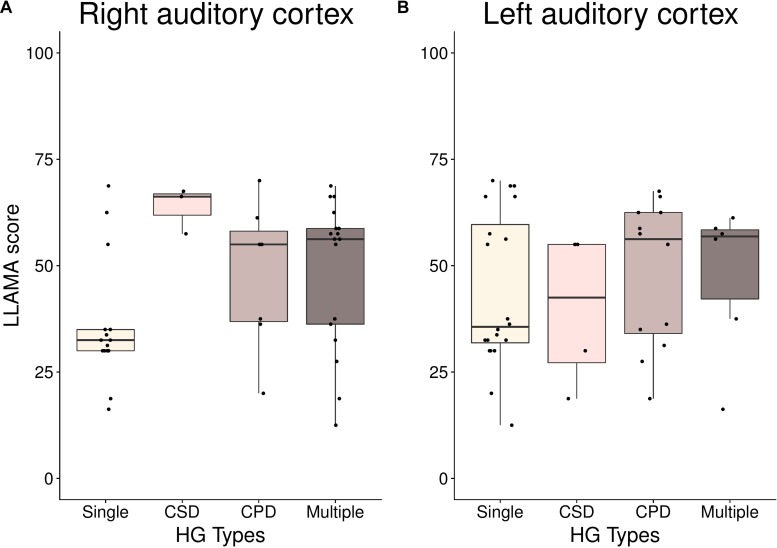
Language aptitude scores (LLAMA; range: 0–100) for different HG types in **(A)** right and **(B)** left AC. From left to right: single gyrus (single), common-stem duplication (CSD), complete posterior duplication (CPD) and multiple gyri (multiple).

**FIGURE 5:**
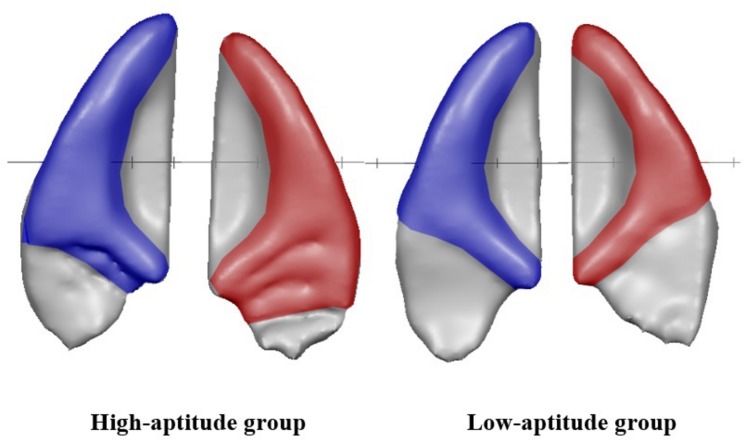
Averaged HG surfaces of high-aptitude group (left panel; *N* = 21) and low-aptitude group (right panel; *N* = 21). Left hemisphere: blue; right hemisphere: red.

To investigate the relationship between the four right-hemispheric HG types and language aptitude, frequency distributions were compared for high-aptitude and low-aptitude subjects according to the LLAMA score. HG types were significantly differently distributed in the two groups (χ^2^_df = 3__,_ *_N_*_= 42_ = 10.01, *p* = 0.012), with multiple gyri being an indicator for high aptitude, and single gyri being an indicator for low aptitude (χ^2^_df = 3__,_ *_N_*_= 42_ = 8.4, *p* = 0.009; 57% of low aptitude children possessing a single gyrus in right AC compared to 14.3% of the children in the high aptitude group). The same tests applied to the left hemisphere yielded no statistically significant results. A comparison of HG types of right and left AC and their association with results of the LLAMA test (mean score) are displayed in Figure 4. A visualization of average surfaces of AC in the right and left hemisphere according to group assignment is illustrated in Figure 5.

We further compared right- and left-hemispheric GM volumes between the two aptitude groups. A Mann–Whitney *U*-test revealed that GM volume in right AC differed significantly between the low- and high-aptitude group [*U_(__40__)_* = 78.00, *Z* = −3.59, *p* < 0.001]. The same test applied to GM volume in left HG yielded no statistically significant results (*U_(__40__)_* = 149.00, *Z* = −1.8, *n.s.*).

This was further corroborated by correlational analyses, which revealed that right, but not left HG GM volume, was associated with a variety of language-relevant variables assessed in our study. These included the number of spoken foreign languages (*r* = 0.35, *p* = 0.022), self-reported language aptitude (*r* = 0.36, *p* = 0.019), parent-reported language aptitude (*r* = 0.38, *p* = 0.024), school grade in German (*r* = 0.46, *p* = 0.003), Hindi speech imitation score (*r* = 0.39, *p* = 0.011), and scores on LLAMA B (*r* = 0.34, *p* = 0.028), LLAMA E (*r* = 0.39, *p* = 0.010), LLAMA F (*r* = 0.45, *p* = 0.003). For LLAMA D there was at least a corresponding non-significant trend (*r* = 0.28, *p* = 0.069). Moreover, a correlation was found for the working memory measure digit span forward (*r* = 0.38, *p* = 0.015).

**FIGURE 6:**
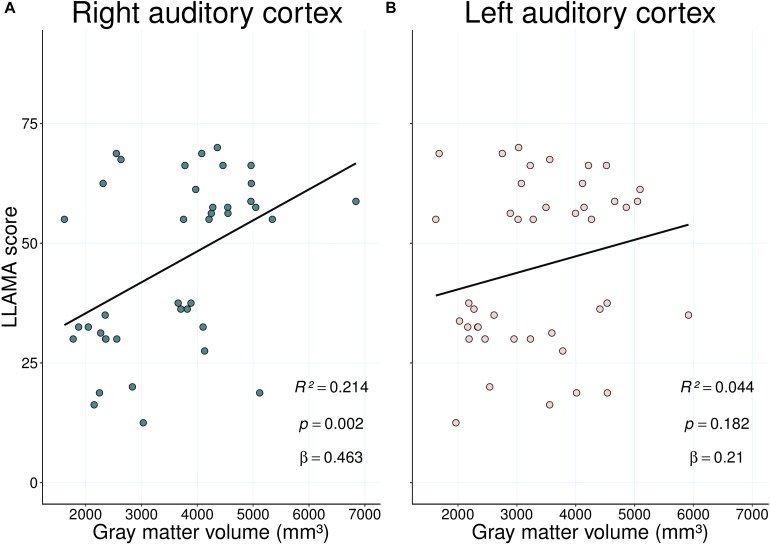
Results of the linear regression showing the relationship between LLAMA mean scores and gray matter volumes (mm^3^) in right **(A)** and left **(B)** AC for all tested individuals. 21.4% of variance in language aptitude are explained by right hemispheric HG gray matter volume.

In addition, we performed linear regression analyses to investigate to what extent the LLAMA overall score predicted left and right GM volumes, respectively (Figure 6). The prediction was only significant for the right HG, where 21.4% of variance in GM volume were explained by language aptitude.

## Discussion

The findings of the present study suggest that the individual neuroanatomy of right AC is significantly associated with language aptitude. In particular, more complete HGs in right AC and higher corresponding GM volumes may be seen as a neuroanatomical marker for high language learning aptitude. Low language aptitude, on the other hand, was significantly linked to the occurrence of single gyri in right AC. Additionally, the intricate relationship between working memory and language could be confirmed through the application of various statistical tests, supporting the strong involvement of working memory in foreign language learning. While also arithmetic skills and school achievement were strongly linked to language aptitude, no relationship between musical ability and language aptitude could be found in the present study.

### Auditory Cortex Morphology as a Marker for Language Aptitude

In accordance with the results of our previous study (Turker et al., 2017), we suggest that the morphology and GM volume of right HG are potential neuroanatomical markers of foreign language aptitude in children and teenagers. These findings also highlight the importance of right-hemispheric auditory processing for language learning (see Kepinska et al., 2017a,b,c) and call for more extensive research on the involvement of AC in foreign language learning.

We argue that AC morphology, both in the form of GM volumes and morphological characteristics, is a stable trait that can be seen as a potential marker for foreign language aptitude. The extent to which interindividual variation in AC morphology is a result of experience-dependent, intrauterine, or genetic influences or a combination thereof, has been discussed in both cross-sectional (Golestani et al., 2011; Herholz and Zatorre, 2012; Ressel et al., 2012; Zatorre, 2013) and longitudinal studies (Hyde et al., 2009; Penhune, 2011), with mixed results so far. Studies with monozygotic and dizygotic twins have demonstrated that morphometric differences of AC are predominantly attributable to genetic factors (Pol et al., 2006). Although musical training has been shown to lead to structural changes (white/GM) after more than a year of training in childhood already (Hyde et al., 2009), results of our own longitudinal studies with children and teenagers (Seither-Preisler et al., 2014; Serrallach et al., 2016) could not corroborate these findings. Our own studies have shown a high inter-individual variability of HG morphology and GM volumes, but an almost perfect intra-individual stability over years, regardless of interim auditory and musical training. Although clear learning-induced changes were established on the neurofunctional level over time, these were not reflected on the neuroanatomical level at all. Thus, we argue that AC morphology and GM volume are highly stable neuroanatomical characteristics that could represent potential markers of aptitude in different auditory-related cognitive domains.

The right hemisphere, and in particular right AC, is implicated in various music-related processes, while studies on language learning so far have found little evidence for involvement of right AC in language learning processes. In our own previous studies we found that right-hemispheric HG morphology and GM volumes were closely linked to musical aptitude (Seither-Preisler et al., 2014; Serrallach et al., 2016), which in turn predicted the motivation to practice a musical instrument. In other words, the larger GM volumes children had, the higher their intrinsic motivation to musically engage. These findings motivated the search for potential neuroanatomical markers for language learning, which are also highly reliant on auditory processing and integration. A subsequent study with German monolingually raised adults (Turker et al., 2017) showed clear evidence that neuroanatomical variation of right AC was linked to higher linguistic abilities. This, in turn, was the primary motivation for the current study in children and teenagers. While in our previous study (Turker et al., 2017) we found a correlation with speech imitation only, the current study extends this finding to language learning on a more general level. In the present study, a strong link was established between a high potential for learning foreign languages both in the receptive and productive domain and the occurrence of multiple HGs in right AC, which was also associated with relatively higher GM volumes. The claim that AC morphology might play a role in foreign language learning has been rarely discussed, in particular since only few studies (Golestani et al., 2007, 2011; Turker et al., 2017) have explicitly addressed a link between the two. This may be because left-hemispheric language dominance has been a well-established fact for decades, which might have discouraged extensive research in language-relevant right-hemispheric brain areas and functions.

The significance of right AC for foreign language learning is also supported by findings of the involvement of right AC in first language acquisition. Perani et al. (2011) found a preponderance of right primary and secondary auditory areas over left AC for speech input in infants, while Dehaene-Lambertz et al. (2006, 2008) found a leftward asymmetry for speech-like stimuli from birth on. Homae et al. (2007) emphasize that speech prosody is one of the most important sources of information for infants in acquiring their native language. They have provided evidence that speech processing in the infant brain develops from analyzing pitch information to comparing and integrating information in input speech sounds with acquired prosodic structures. According to their neuroimaging findings in 10-month old infants, cortical activation in response to manipulated speech prosody was clearly right lateralized in temporal and temporo-parietal regions. This suggests that in early infancy right-hemispheric auditory functions are essential and predominant, until more refined left-hemispheric language comprehension skills step into the foreground. This is also consistent with findings of an earlier fetal and postnatal maturation of the right hemisphere in general, which enables global feature extraction before more complex analytic skills become predominant (for a review see Chiron et al., 1997). As right-hemispheric auditory processing is focused on the recognition of vocal timbre and prosody, it is likely that right-hemispheric brain areas play a crucial role in early native language acquisition by infants. Moreover, they should be relevant for the ability to learn new languages in later life, which also requires the recognition and memorization the prosodic contours of the unfamiliar language before explicitly using and manipulating its elements. Therefore, an advantageous neuroanatomical morphology of relevant areas in right AC and corresponding large GM volumes could consequently be associated with high language aptitude.

Given the potential involvement of right AC morphology in foreign language aptitude, it seems worthwhile to look at the various functions of primary and secondary auditory areas in the processing of speech. HG includes a posteromedial primary and an anterolateral secondary part and also shows a characteristic hemispheric specialization (Zoellner et al., 2018). While primary auditory regions are crucial for the analysis of simple sound features, secondary areas enable a larger integration and hence more complex auditory pattern recognition, relevant for the processing of music and speech (Leaver and Rauschecker, 2010). There have been numerous studies addressing the functional lateralization of AC (Schirmer et al., 2012). Different studies have provided evidence that the left AC possesses a sensitivity for the processing of fast acoustic events (e.g., speech; Zatorre et al., 2002), while the right AC is specialized for finer resolution in the frequency domain (Zatorre et al., 2002; Poeppel, 2003; Hyde et al., 2008). Concerning HG, the left side seems to be more sensitive to the temporally conveyed fundamental pitch of complex tones, while the right side seems to be more important for the spectral discrimination of different timbres of musical and vocal sounds (Schneider et al., 2005). It has also been suggested that left and right AC have distinct temporal integration windows with the left being specialized in the analysis of short segments (25–50 ms) and the right in long segments (200–300 ms; Poeppel, 2001; Boemio et al., 2005; Liem et al., 2014). That predisposes right AC not only to analyze slow musical rhythms and melodic contours, but also to process the syllabic structure and prosodic modulations of spoken language (Leong and Goswami, 2014), explaining bilateral involvement of AC in syllabic processing (Sheng et al., 2018). In addition, there is evidence that lateral right AC is specialized in spatial sound processing (Zatorre and Penhune, 2001). In our previous studies, we found clear evidence that an efficient integration of these left and right-hemispheric auditory functions promotes phonological awareness, literacy skills and attention (Seither-Preisler et al., 2014; Serrallach et al., 2016). Each AC is a hub for numerous neural circuits to other brain areas that are involved in multimodal processing, motor activation and speech production (Friederici and Gierhan, 2013). Having more efficient and faster processing and integration of speech units (be it single sounds, syllables or whole words) could definitely prove advantageous for foreign language learning. Higher GM volumes in certain parts of AC might provide better structural links to other language-relevant areas, e.g., the inferior parietal cortex, and prove advantageous for functional connectivity. To summarize, variations in the individual neuroanatomy of specific brain regions, such as HG or AC more generally, should not be neglected and could be used to further develop theories on language learning and aptitude.

In sum, the neuroanatomical results of the present study confirm our hypotheses that foreign language aptitude is significantly associated with right hemispheric AC morphology and GM volumes. In our study, multiple gyri and larger GM volumes of HG in right AC turned out to be correlated with high language aptitude both in the receptive and productive domain. Single gyri in right AC, on the other hand, were significantly linked to low language aptitude. This is consistent with our own previous study (Turker et al., 2017) and also in accordance with studies of Kepinska et al. (2017a,b,c, 2018), highlighting the right-hemispheric involvement in initial stages of learning a foreign language.

### The Link Between Language Aptitude, Working Memory and Arithmetic Competence

Researchers have argued that language aptitude or high language aptitude and working memory are so intricately linked that working memory capacity equals outstanding aptitude or at least deserves to be seen as a dominant sub-component (Wen, 2012; Wen et al., 2017). Our study supports this hypothesis in so far that language aptitude scores and working memory scores highly correlated with one another. This was also reflected in the results of the PCA, which indicated that working memory and language aptitude loaded onto one single factor. Thus, working memory appears to be a crucial constituent of language aptitude. Both language aptitude and working memory also correlated significantly with the arithmetic score. Obviously, parts of the language aptitude tests and the arithmetic fluency test touch upon working memory and analytic abilities, leading to linked scores in both. This is consistent with earlier results from DeStefano and LeFevre (2004) and Peng et al. (2015) and suggests that working memory is an essential, shared foundation of arithmetic and language skills.

It has been suggested that the Hindi speech imitation test captures aspects of language aptitude and working memory capacity (see Reiterer et al., 2011). Also in our study, children from the high language aptitude group had significantly higher Hindi speech imitation scores. However, correlational analyses showed only one significant correlation with the subtest LLMA B (vocabulary learning), but not with the three other language aptitude scales. Furthermore, there was a lack of correlation between the Hindi and working memory scores. This is particularly surprising, since one might assume that the Hindi test is a mere non-word span task, which comes in two complexities, namely single words and whole sentences. A possible reason for the observed lack of association is that repetition of real speech material that is either similar or not to the participant’s native language makes a difference, since it is much more complex (full sentences in the Hindi task, combinations of simple CV syllables in the non-word span task). This might require skills that go beyond simple working memory capacity and also require complex auditory pattern recognition and generalization of common language-specific acoustic features.

### Behavioral Findings: School Grades, Self-Rated and Parent-Reported Aptitude

Children who scored significantly better in the language aptitude testing also had better school grades in both English and German, were learning more languages at the time of testing and thought of themselves as better language learners. The positive relationship between the number of foreign languages being learnt and the language aptitude scores can be explained by two theories: (1) Children who learn languages quite effortlessly and fast know about their gift through the ease with which they have learnt English, for instance, and therefore they chose to learn more foreign languages (whether at school or through extracurricular activities); (2) Children who have learnt more languages could have profited from the language classes in so far that their meta-linguistic awareness is particularly higher and therefore they score better in the language aptitude testing. Although no causal evidence can be gained through correlational analyses in the present study, we consider both possibilities good explanations for the observed correlations.

Certainly, school grades are not an optimal indicator for real potential, but one would assume that the ease with which foreign languages are learnt (by remembering vocabulary, understanding grammatical complexity) should be at least reflected in those grades. It furthermore seems that both parents and children have a very good intuition regarding their child’s/their own language learning potential and this intuition is well supported by their scores in the language aptitude batteries. This self-perceived aptitude might impact school grades as well, since more confidence could boost motivation and thereby influence behavior at school in language classes.

## Conclusion

In accordance with our previous study, we could verify the importance of right-hemispheric AC morphology for foreign language aptitude. A higher number of HGs and corresponding higher GM volumes in right AC were associated with higher performance in the LLAMA language aptitude battery, while single gyri were significantly associated with low performance on the language aptitude tests. Moreover, language aptitude showed a strong link with working memory capacity, speech imitation skills and arithmetic abilities. Those children with high language aptitude also had better school grades in English, German, considered themselves more gifted for language learning and were estimated to be better language learners by their parents. The behavioral findings of this study suggest that language aptitude is associated with working memory and arithmetic abilities and impacts school performance and self-perception, as well as parents’ opinions of their children. In contrast to earlier studies, no association between musicality and language aptitude could be found.

## Ethics Statement

This study was carried out in accordance with the recommendations of the ethics committee of the Heidelberg Medical School (votum S 616/2015).

## Author Contributions

ST, SR, and PS designed the study, and acquired, analyzed, and interpreted the data. ST and AS-P wrote the manuscript. AS-P and ST performed the statistical analyses. SR and PS provided assistance with the manuscript in the form of comments and feedback.

## Conflict of Interest Statement

The authors declare that the research was conducted in the absence of any commercial or financial relationships that could be construed as a potential conflict of interest.
